# Ischemic acute pancreatitis with pancreatic pseudocyst in a patient with abdominal aortic aneurysm and generalized atheromatosis – case report

**DOI:** 10.1186/s12876-015-0258-6

**Published:** 2015-03-21

**Authors:** Ileana Cocota, Radu Badea, Traian Scridon, Dan L Dumitrascu

**Affiliations:** 1 2nd Medical Department, Cluj-Napoca, Romania; 2 Department of Clinical Imaging Ultrasound, “Iuliu Hatieganu” University of Medicine and Pharmacy, Cluj-Napoca, Romania; 3 Heart Institute “Prof. Dr. Nicolae Stancioiu”, “Iuliu Hatieganu” University of Medicine and Pharmacy, Cluj-Napoca, Romania; 4 2nd Medical Department, “Iuliu Hatieganu” University of Medicine and Pharmacy, Cluj-Napoca, Romania

**Keywords:** Ischemic pancreatitis, Pancreatic pseudocyst, Abdominal aortic aneurysm, Atherosclerosis

## Abstract

**Background:**

Ischemic pancreatitis is a rare medical entity. The pancreatic tissue is susceptible to ischemia with the possibility of developing acute pancreatitis. The abdominal aortic aneurysm can be one possible cause of pancreatic hypoperfusion.

**Case presentation:**

We report the case of a 68-year-old man, Caucasian, with a history of a cluster of severe cardiovascular conditions, who presented epigastric pain of variable intensity for about 2 weeks. The pain occurred after intense physical effort, and was associated with anorexia and asthenia. The palpation revealed epigastric pain and palpable pulsatile mass above the umbilicus. Laboratory tests showed increased serum and urine amylases. The abdominal contrast-enhanced CT scan evidenced acute lesions of the pancreas and a caudal pancreatic pseudocyst of 39x24 mm. An abdominal aortic aneurysm was also described (which extended from the kidney level to the bilateral femoral level) with a maximum diameter of 60.5 mm and generalized atheromatosis. By corroborating clinical, anamnestic, laboratory and imaging data, the case was diagnosed as moderately severe acute ischemic pancreatitis, pancreatic pseudocyst, abdominal aortic aneurysm, generalized atheromatosis. The pancreatic pseudocyst was resorbed in eight months. Surgery for the abdominal aneurysm was performed after the resorption of the pseudocyst. The patient died after aortic surgery because of a septic complication.

**Conclusion:**

Ischemic pancreatitis is a rare condition but should be considered in a patient with upper abdominal pain and elevated amylase in the context of an abdominal aortic aneurysm and generalized atheromatosis.

## Background

The most frequent etiological factors of acute pancreatitis are gallstone disease and alcohol consumption, followed by hyperlipemia, pancreatic malformations, autoimmunity, etc. [1]. Pancreatic ischemia is a rare cause for acute pancreatitis and in most cases it develops only into mild or moderate forms, but there are also forms with extensive pancreatic necrosis that can progress to pancreatic abscesses [1]. Ischemic acute pancreatitis should be treated as any other form of acute pancreatitis, removing, of course, the cause of pancreatic hypoperfusion whenever possible.

## Case presentation

We present the case of a 68-year-old male patient, Caucasian, living in an urban environment, who was admitted in emergency for epigastric pain. His personal history included duodenal ulcer 20 years ago, coronary heart disease, heart failure diagnosed 6 years ago, with PTCA and stent implantation in the anterior interventricular branch of the left coronary artery 5 years ago, exertional angina pectoris, dilated cardiomyopathy, hypertension risk grade III, hypertensive cardiomyopathy, type 2 diabetes (controlled by oral therapy). The epigastric pain had set on suddenly 2 weeks before, after an intense physical effort (and with inadequate hydration) and radiated to the back. The pain had variable intensity during its evolution, initially described as being of great intensity, later as having an average intensity and was accompanied by anorexia and asthenia. The patient reported associated progressive exertional dyspnea and chest pain with angina characteristics when making intensive efforts.

The patient denied any history of gallstone disease and any high-fat, high-calorie intake or alcohol consumption. He was on a therapeutic regimen containing angiotensin-converting enzyme inhibitor (Prestarium 5 mg once daily), beta blocker (Carvedilol 6,25 mg twice daily), antiplatelet (Aspirin 75 mg once daily), nitrate (Isosorbide mononitrate 40 mg once daily), statin (Rosuvastatin 10 mg once daily) and biguanide (Metformin 800 mg once daily); was compliant to this therapy. As a risk factor he was an ex-smoker, but did not present any familial risk factor.

Clinical findings on hospital admission: apyrexia; asthenia; no pulmonary findings; rhythmic heart sounds, heart rate = 56 beats/min, blood pressure = 140/85 mmHg; no peripheral swelling; abdomen with tenderness in the epigastric area and palpable pulsatile mass above the umbilicus, no defense or muscle stiffness, Murphy and Blumberg signs were negative; normal bowel movements; the diuresis was normal.

The blood tests showed: ESR = 20 mm/h, normal blood count, increased serum (158 UI/l, 1.5 × normal) and urinary amylase (1053 UI/l, 2 × normal), serum glucose = 119 mg/dl, hepatic and renal functions unaltered, normal triglycerides and cholesterol.

The chest x-ray showed no pathological changes.

**Figure 1:**
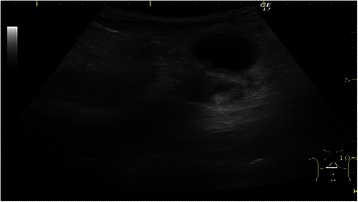
**Abdominal ultrasound: pancreatic pseudocyst.**

**Figure 2:**
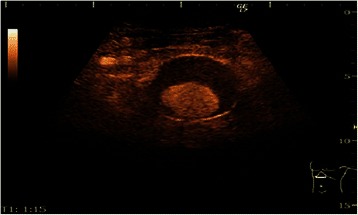
**Abdominal contrast-enhanced ultrasound: abdominal aortic aneurysm.**

The abdominal ultrasound followed by abdominal contrast-enhanced ultrasound (CEUS) revealed a 40 mm pancreatic pseudocyst with sediment (Figure 1) and the dilation of an abdominal aortic aneurysm (diameter > 55 mm), (Figure 2), liver steatosis, no gallstones, spleen and kidneys with normal ultrasound appearance.

**Figure 3:**
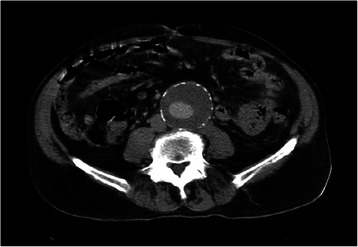
**Abdominal CT with contrast: partially thrombosed aneurysm of the abdominal aorta.**

The CT examination with contrast substance (Ioperamidum) described the spindle-shaped aneurysm of the abdominal aorta dilation beginning from the renal level, extending to 169 mm, until the iliac bifurcation and continuing on both iliac arteries, 40 mm left and 20 mm right respectively. The maximum diameter of the aortic aneurysm was 60.5 mm, with calcified circumferential atheromatosis, diffuse on its whole length; a parietal circumferential thrombosis with a maximum thickness of 27 mm and a minimum of 7 mm was also present; the diameter of the circulated lumen was 19 mm in the distal portion of the aorta, and 29/32 mm respectively below the kidneys. At the level of the renal arteries the aorta had an antero-posterior caliber of 36 mm and a latero-lateral diameter of 42 mm, with thrombosis of 5 mm postero-laterally. No signs of dissection or rupture of the aneurysm were evidenced. Calcified atheromas were described at the level of the splenic artery, and also at the level of the superior mesenteric artery ostium and bilateral renal arteries (Figure 3). On suprarenal and throracic sections, the aorta had a normal caliber.

**Figure 4:**
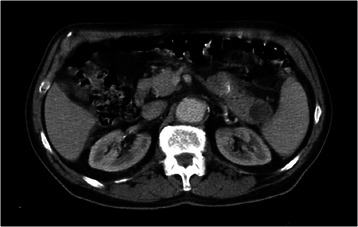
**Abdominal CT with contrast: caudal pancreatic pseudocyst.**

The CT examination with contrast substance highlighted a round-oval lesion of 39/24 mm, with a slightly heterogeneous structure, partially with fluid content, at the level of the pancreas tail. In the omental bursa and near the tail of the pancreas there was fluid collection and fatty infiltration. Otherwise, the pancreas had a normal aspect, and the duct of Wirsung was not dilated. The remaining abdominal organs were normal on the CT scan. (Figure 4). Based on the clinical and anamnestic data (patient denied alcohol consumption or a high fat intake; gallstones were absent; pain appeared after an intensive physical effort), together with the laboratory and imaging data, the case was interpreted as moderately severe ischemic acute pancreatitis [2], pancreatic pseudocyst, partially thrombosed aneurysm of the abdominal aorta, general atheromatosis.

Taking into account the presence of ischemic acute pancreatitis and partially thrombosed aortic aneurysm, a superior gastrointestinal endoscopy was recommended in order to exclude ischemic gastropathy but the patient refused to undergo this procedure.

The cardiovascular surgery examination and the general surgery consultation recommended the abdominal aortic aneurysm surgery to be postponed for 2–3 months, waiting for spontaneous resolution of the pancreatic pseudocyst.

The patient followed a standard therapy including dietary restrictions, hydroelectrolytic rebalancing, proton pump inhibitors, antispasmodics, antibiotics, pancreatic enzymes together with the cardiologic treatment (angiotensin-converting-enzyme inhibitor, beta blockers, antiplatelet therapy, nitrates, statin) and oral antidiabetic drugs. The evolution was favorable with slow normalization of pancreatic enzymes.

**Figure 5:**
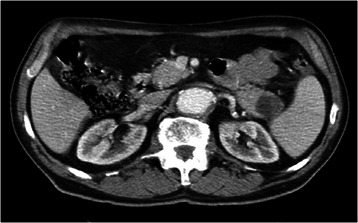
**Abdominal CT with contrast: caudal pancreatic pseudocyst (after 2 months).**

After 2 months, the patient returned for follow-up examination. His clinical condition was improved, he did not present abdominal tenderness. Biological findings: ESR = 20 mm/h; normal pancreatic enzymes. The repeated abdominal contrast CT scan showed no significant changes in size for the pancreatic pseudocyst and the abdominal aortic aneurysm (Figure 5).

This time gastroscopy was performed, showing no pathological changes.

**Figure 6:**
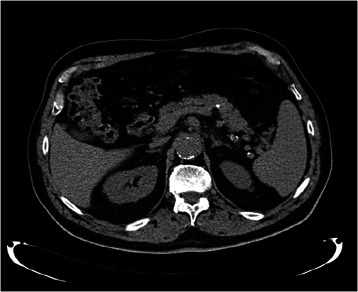
**Abdominal CT native: normal pancreatic structure (after eight months).**

A cardiovascular surgery consultation was performed again, and the patient was scheduled for aortobifemoral endoluminal prosthesis. Meanwhile the follow–up showed that the pancreatic pseudocyst had resorbed in eight months. (Figure 6). Surgery was delayed due to an intercurrent respiratory infection of the patient (bronchitis).

Surgery for the abdominal aneurysm was performed after the resorption of the pseudocyst. The cure of the aortic aneurysm was performed by bypass grafting with dacron aorto-bifemoral prosthesis. After surgery, a complication appeared, namely a periprosthetic retroperitoneal abscess. Another surgical intervention was therefore necessary, but severe septic shock occurred, leading to the patient’s death.

## Discussion

The arterial vascularization of the pancreas is ensured by branches of the hepatic artery and superior mesenteric artery for the head of the pancreas, and branches from the splenic artery for the body and the tail of the pancreas. It is known that the pancreatic tissue is susceptible to ischemia with the possibility of developing acute pancreatitis [1]. The appearance of oxygen-derived free radicals that affect the microvascularization, leading to endothelial dysfunction, increase of the permeability for active proteases, affecting intracellular homeostasis, as well as activating the polymorphonuclear cells, represent important mechanisms in the pathogenesis of ischemic pancreatitis [3]. Ischemic acute pancreatitis may have different presentations: in some cases it can be expressed as a prolonged increase of serum and urinary amylases, accompanied by minimal symptoms, which in most cases are solved spontaneously; but in some cases complications may occur, i.e. pancreatic necrosis and abscess formation [1].

Cases of acute pancreatitis after cardiogenic shock determined by cardiac tamponade have been described [4], supported also by postmortem studies [5] or studies on experimental animals [6]. Cases of ischemic acute pancreatitis after cardiac arrest followed by reversible resuscitation [7], but also as a complication of intra-aortic balloon counterpulsation have also been published [8]. Ischemic pancreatitis after aortic dissection of an abdominal aortic aneurysm has been reported [9,10], and even as a rare complication of surgical treatment of thoraco-abdominal aortic aneurysm [11-13].

Generalized atheromatosis with significant impairment of splanchnic circulation was also considered a cause, which determines the appearance of pancreatic necrosis of an ischemic type [11]. A case of acute ischemic pancreatitis was reported in an individual who had performed intense physical exercise without appropriate fluid intake [14].

Our patient – in the context of the partially thrombosed aneurysm of the abdominal aorta (which extended from kidney level to femoral arteries) and generalized atheromatosis (with calcified, raw atheroma, at the level of the splenic artery, superior mesenteric artery ostium and renal arteries), he had undertaken intense physical effort, with an increased need for oxygen at the level of the muscles, to the detriment of the splanchnic territory, i.e. pancreatic, causing the onset of ischemic acute pancreatitis. We have also excluded any other possible causes of pancreatitis. In the case described, the pathogenetic mechanism of pancreatitis is explained by the arterial steal phenomenon, aneurysm of the aorta causing pancreatic hypoperfusion, especially in the presence of generalized atheromatosis.

Because the surgical treatment of an aortic aneurysm itself can suffer complications with the appearance of pancreatitis of an ischemic type, the intervention was delayed until the normalization of pancreatic enzymes and the limitation of the lesions. Surgery for the abdominal aneurysm was performed after the resorption of the pseudocyst. But postoperative complications occurred - periprosthetic retroperitoneal abscess and severe septic shock, which led to the patient’s death.

This case has several particular features. The diagnosis of a partially thrombosed aneurysm of the abdominal aorta could be established only after the presentation for abdominal pain caused by ischemic recurrent acute pancreatitis, i.e. after an unusual complication. Both the thrombosed aneurysm of the abdominal aorta and the generalized atheromatosis were simultaneously detected, which significantly affected the pancreatic circulation, each of these entities being able to cause ischemic pancreatitis. It should be mentioned that even the surgery for abdominal aortic aneurysm itself may cause pancreatic ischemia and an ischemic episode of pancreatitis; therefore, aorto-bifemoral endoluminal prosthesis was delayed until the normalization of serum pancreatic enzymes.

## Conclusions

Ischemic pancreatitis is a rare condition but should be taken into consideration for patients with upper abdominal pain, elevated amylases and significant cardiovascular diseases with extended atheromatosis. The diagnosis relies on the exclusion of other more common causes of pancreatitis.

## Consent

Written informed consent was obtained from the patient’s family for the publication of this case report. A copy of the written consent is available for review by the Editor-in-Chief of this journal.

## Abbreviations

PTCA: Percutaneous transluminal coronary angioplasty
ESR: Erythrocyte sedimentation rate
CT: Computed tomography
CEUS: Abdominal contrast-enhanced ultrasound
